# Supplementary material for: Receptor-informed network control theory links LSD and psilocybin to a flattening of the brain’s control energy landscape
Source: Nat Commun. 2022 Oct 3;13:5812. doi: 10.1038/s41467-022-33578-1 (PMC9530221; doi:10.1038/s41467-022-33578-1)
Supplement: Supplementary file 3 — Reporting Summary [file 41467_2022_33578_MOESM3_ESM.pdf]

nature portfolio

Corresponding author(s): S. Parker Singleton

Last updated by author(s): 08/08/2022

Reporting Summary

Nature Portfolio wishes to improve the reproducibility of the work that we publish. This form provides structure for consistency and transparency in reporting. For further information on Nature Portfolio policies, see our Editorial Policies and the Editorial Policy Checklist.

Statistics

For all statistical analyses, confirm that the following items are present in the figure legend, table legend, main text, or Methods section.

n/a	Confirmed	
☐	☒	The exact sample size (n) for each experimental group/condition, given as a discrete number and unit of measurement
☐	☒	A statement on whether measurements were taken from distinct samples or whether the same sample was measured repeatedly
☐	☒	The statistical test(s) used AND whether they are one- or two-sided<br>*Only common tests should be described solely by name; describe more complex techniques in the Methods section.*
☐	☒	A description of all covariates tested
☐	☒	A description of any assumptions or corrections, such as tests of normality and adjustment for multiple comparisons
☐	☒	A full description of the statistical parameters including central tendency (e.g. means) or other basic estimates (e.g. regression coefficient) AND variation (e.g. standard deviation) or associated estimates of uncertainty (e.g. confidence intervals)
☐	☒	For null hypothesis testing, the test statistic (e.g. F, t, r) with confidence intervals, effect sizes, degrees of freedom and P value noted<br>*Give P values as exact values whenever suitable.*
☒	☐	For Bayesian analysis, information on the choice of priors and Markov chain Monte Carlo settings
☒	☐	For hierarchical and complex designs, identification of the appropriate level for tests and full reporting of outcomes
☐	☒	Estimates of effect sizes (e.g. Cohen's d, Pearson's r), indicating how they were calculated

Our web collection on statistics for biologists contains articles on many of the points above.

Software and code

Policy information about availability of computer code

Data collection	Data collection and preprocessing has been reported previously (Carhart-Harris et al 2016, PNAS). No software was used in the data collection.
Data analysis	MATLAB R2017a or later. R 3.2.5 or later. See "Preprocessing Software" section for software used in preprocessing. Code is available at: 10.5281/zenodo.6968138

For manuscripts utilizing custom algorithms or software that are central to the research but not yet described in published literature, software must be made available to editors and reviewers. We strongly encourage code deposition in a community repository (e.g. GitHub). See the Nature Portfolio guidelines for submitting code & software for further information.

Data

Policy information about availability of data

All manuscripts must include a data availability statement. This statement should provide the following information, where applicable:

- Accession codes, unique identifiers, or web links for publicly available datasets
- A description of any restrictions on data availability
- For clinical datasets or third party data, please ensure that the statement adheres to our policy

The fMRI LSD data are freely available at https://openneuro.org/datasets/ds003059/versions/1.0.0. The voxelwise receptor density maps are freely available at: https://xtra.nru.dk/FS5ht-atlas/. All other functional and structural data, along with parcellated receptor maps, and the source data to reproduce the main figures are available on our Zenodo repository (10.5281/zenodo.6968138).

Field-specific reporting

Please select the one below that is the best fit for your research. If you are not sure, read the appropriate sections before making your selection.

- [x] Life sciences
- [ ] Behavioural & social sciences
- [ ] Ecological, evolutionary & environmental sciences

For a reference copy of the document with all sections, see nature.com/documents/nr-reporting-summary-flat.pdf

Life sciences study design

All studies must disclose on these points even when the disclosure is negative.

Sample size	Initial sample size was 20 subjects for LSD study and 15 for psilocybin. The sample sizes are in line with previous fMRI studies involving pharmacological interventions with controlled substances and were restricted by a scarcity of funding.
Data exclusions	LSD: 1 subject exited the scanner early due to anxiety and 4 subjects' fMRI scans were discarded due to head motion. Psilocybin: 6 were discarded due to excessive head motion (>15% scrubbed volumes when the scrubbing threshold is FD = 0.5)
Replication	Each subject one only scanned once per condition (once under drug and once under placebo; approximately two weeks apart for LSD and one week for psilocybin).
Randomization	On one day, the participants received placebo, and on the other day they received LSD/psilocybin. The order of the conditions was balanced across participants.
Blinding	Participants were blind to this order (above) but the researchers were not for safety purposes.

Reporting for specific materials, systems and methods

We require information from authors about some types of materials, experimental systems and methods used in many studies. Here, indicate whether each material, system or method listed is relevant to your study. If you are not sure if a list item applies to your research, read the appropriate section before selecting a response.

Materials & experimental systems

n/a	Involved in the study	
x		Antibodies
x		Eukaryotic cell lines
x		Palaeontology and archaeology
x		Animals and other organisms
	x	Human research participants
x		Clinical data
x		Dual use research of concern

Methods

n/a	Involved in the study	
x		ChIP-seq
x		Flow cytometry
	x	MRI-based neuroimaging

Human research participants

Policy information about studies involving human research participants

Population characteristics	Exclusion criteria included: < 21 years of age, personal history of diagnosed psychiatric illness, immediate family history of a psychotic disorder, an absence of previous experience with a classic psychedelic drug (e.g. LSD, mescaline, psilocybin/magic mushrooms or DMT/ayahuasca), any psychedelic drug use within 6 weeks of the first scanning day, pregnancy, problematic alcohol use (i.e. > 40 units consumed per week), or a medically significant condition rendering the volunteer unsuitable for the study.<br><br>Out of the 20 subjects who participated in the LSD study, 15 were found suitable for analysis (four females; mean age, 30.5 ± 8.0 y). Out of the 15 who participated in the psilocybin study, 9 were found suitable for analysis (two females; age, 32 ± 8.9 SD y of age).
Recruitment	All participants were recruited via word of mouth and provided written informed consent to participate after study briefing and screening for physical and mental health
Ethics oversight	This study was approved by the National Research Ethics Service committee London-West London and was conducted in accordance with the revised declaration of Helsinki (2000), the International Committee on Harmoniza- tion Good Clinical Practice guidelines, and National Health Service Research Governance Framework. Imperial College London sponsored the research, which was conducted under a Home Office license for research with schedule 1 drugs.

Note that full information on the approval of the study protocol must also be provided in the manuscript.

Magnetic resonance imaging

Experimental design

Design type	Resting-state
Design specifications	LSD: On each visit, BOLD scanning consisted of three eyes-closed resting state scans recorded approximately 2hr after drug/placebo infusion, each lasting seven minutes. The first and third scans were eyes-closed rest but the second scan also incorporated listening to some music. Only the first and third were used for the main analysis, however results were replicated using only the music (second) scans as well. Sessions were recorded at least two weeks apart. Psilocybin: All subjects underwent two 12-min eyes-closed resting-state fMRI scans over separate sessions, at least 7 d apart. In each session, subjects were injected i.v. with either psilocybin or a placebo. The injections were given manually by a medical doctor within the scanning suite. The infusions began exactly 6 min after the start of the 12-min scans and lasted 60 s.
Behavioral performance measures	After each scan, VAS ratings were performed in the scanner via a response-box.

Acquisition

Imaging type(s)	functional
Field strength	3T
Sequence & imaging parameters	LSD: BOLD-weighted fMRI data were acquired using a gradient echo planer imaging sequence, TR/TE = 2000/35ms, field-of-view = 220mm, 64 × 64 acquisition matrix, parallel acceleration factor = 2, 90° flip angle. Thirty five oblique axial slices were acquired in an interleaved fashion, each 3.4mm thick with zero slice gap (3.4mm isotropic voxels). The precise length of each of the two BOLD scans was 7:20 minutes. Psilo: BOLD-weighted fMRI data were acquired at 3T using a gradient echo EPI sequence, TR/TE 3000/35 ms, field-of-view = 192 mm, 64 × 64 acquisition matrix, parallel acceleration factor = 2, 90° flip angle. Fifty-three oblique axial slices were acquired in an interleaved fashion, each 3 mm thick with zero slice gap (3 × 3 × 3-mm voxels).
Area of acquisition	whole brain

Diffusion MRI ☐ Used ☒ Not used

Preprocessing

Preprocessing software	Freesurfer v5.0 or later; AFNI 2015 or later, FMRIB Software Library (FSL) v5.0 or later, Advanced Normalization Tools (ANTS) v2.0 or later.<br><br>LSD: The following pre- processing stages were performed: 1) removal of the first three volumes; 2) de-spiking (3dDespike, AFNI); 3) slice time correction (3dTshift, AFNI); 4) motion correction (3dvolreg, AFNI) by registering each volume to the volume most similar, in the least squares sense, to all others (in-house code); 5) brain extraction (BET, FSL); 6) rigid body registration to anatomical scans (twelve subjects with FSL's BBR, one subject with Freesurfer's bbregister and two subjects manually); 7) non-linear registration to 2mm MNI brain (Symmetric Normalization (SyN), ANTS); 8) scrubbing (8) - using an FD threshold of 0.4 (the mean percentage of volumes scrubbed for placebo and LSD was 0.4 ±0.8% and 1.7 ±2.3%, respectively). The maximum number of scrubbed volumes per scan was 7.1%) and scrubbed volumes were replaced with the mean of the surrounding volumes. Additional pre- processing steps included: 9) spatial smoothing (FWHM) of 6mm (3dBlurInMask, AFNI); 10) band-pass filtering between 0.01 to 0.08 Hz (3dFourier, AFNI); 11) linear and quadratic de-trending (3dDetrend, AFNI); 12) regressing out 9 nuisance regressors (all nuisance regressors were bandpassed filtered with the same filter as in step 10): out of these, 6 were motion-related (3 translations, 3 rotations) and 3 were anatomically-related (not smoothed). Specifically, the anatomical nuisance regressors were: 1) ventricles (Freesurfer, eroded in 2mm space), 2) draining veins (DV) (FSL's CSF minus Freesurfer's Ventricles, eroded in 1mm space) and 3) local white matter (WM) (FSL's WM minus Freesurfer's subcortical grey matter (GM) structures, eroded in 2mm space). Psilo: same as above
Normalization	non-linear registration to 2mm MNI brain (Symmetric Normalization (SyN), ANTS)
Normalization template	MNI 2mm
Noise and artifact removal	LSD: One subject did not complete the BOLD scans due to anxiety and an expressed desire to exit the scanner and four others were discarded from the group analyses due to excessive head movement. Principally, motion was measured using frame-wise displacement (FD) (7). The criterion for exclusion was subjects with >15% scrubbed volumes when the scrubbing threshold is FD = 0.5. After discarding these subjects we reduced the threshold to FD = 0.4. The between-condition difference in mean FD for the 4 subjects that were discarded was 0.323±0.254 and for the 15 subjects that were used in the analysis the difference in mean FD was 0.046 ±0.032. Psilo: same as above.
Volume censoring	above - scrubbed volumes replaced with mean of surrounding volumes

Statistical modeling & inference

Model type and settings	k-means clustering of activation patterns and network control theory

Effect(s) tested | Resting-state LSD/psilo compared to Placebo

Specify type of analysis: ☐ Whole brain ☒ ROI-based ☐ Both

Anatomical location(s) | A whole-brain atlas of 463 regions was used.

Statistic type for inference (See Eklund et al. 2016) | n/a

Correction | Benjamini-Hochberg

Models & analysis

n/a	Involved in the study
☒	☐ Functional and/or effective connectivity
☒	☐ Graph analysis
☐	☒ Multivariate modeling or predictive analysis

Multivariate modeling and predictive analysis | Group comparison of brain-activity profiles and correlations of brain-activity profile and transition energy calculations
